# Supplementary figures and images for: Predictive value of uric acid to albumin ratio for carotid atherosclerosis in type 2 diabetes mellitus: A retrospective study
Source: PLoS One. 2025 Mar 28;20(3):e0320738. doi: 10.1371/journal.pone.0320738 (PMC11952251; doi:10.1371/journal.pone.0320738)

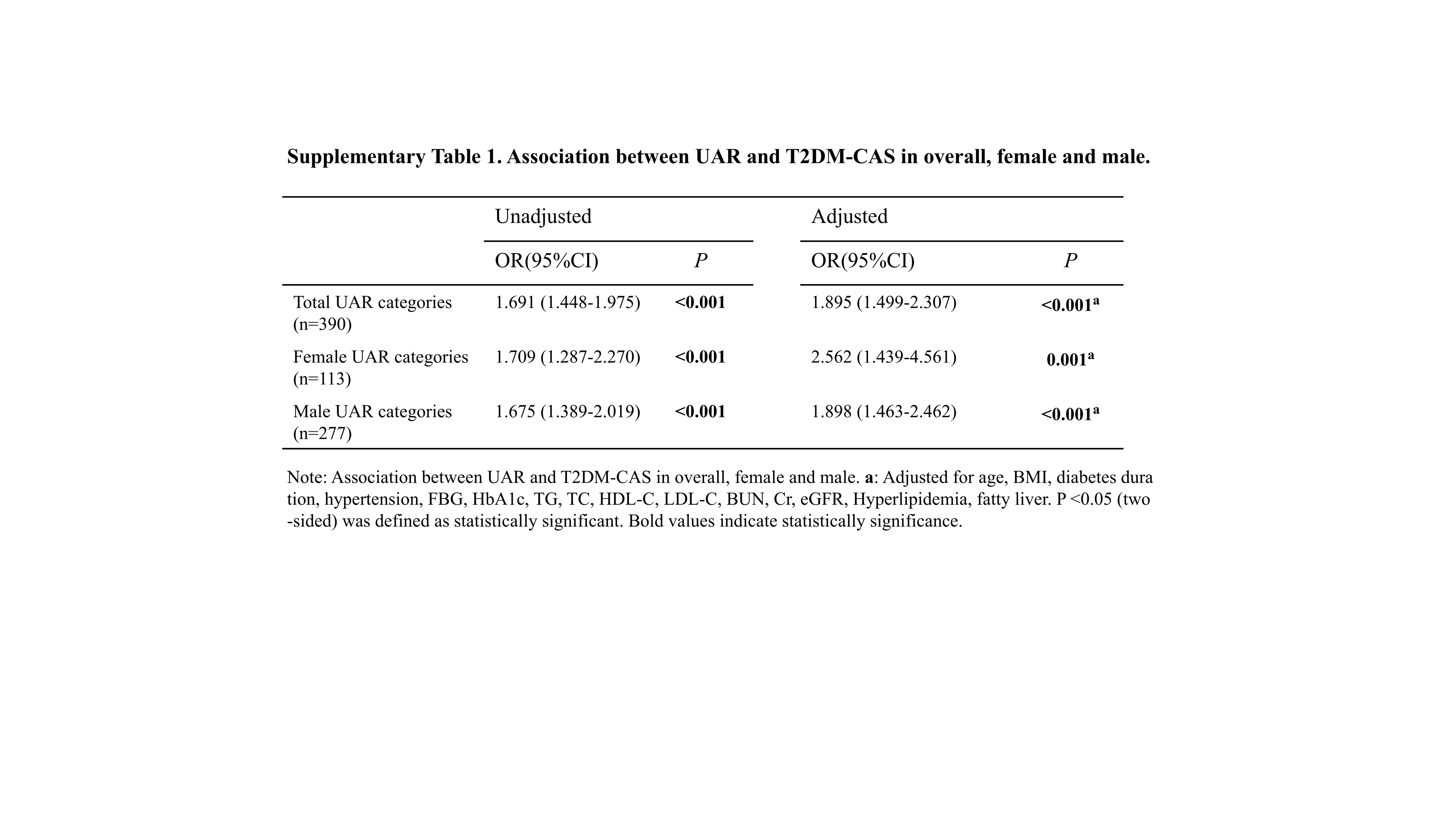

Supplement: S1 Fig — (TIF) [file pone.0320738.s002.tif]

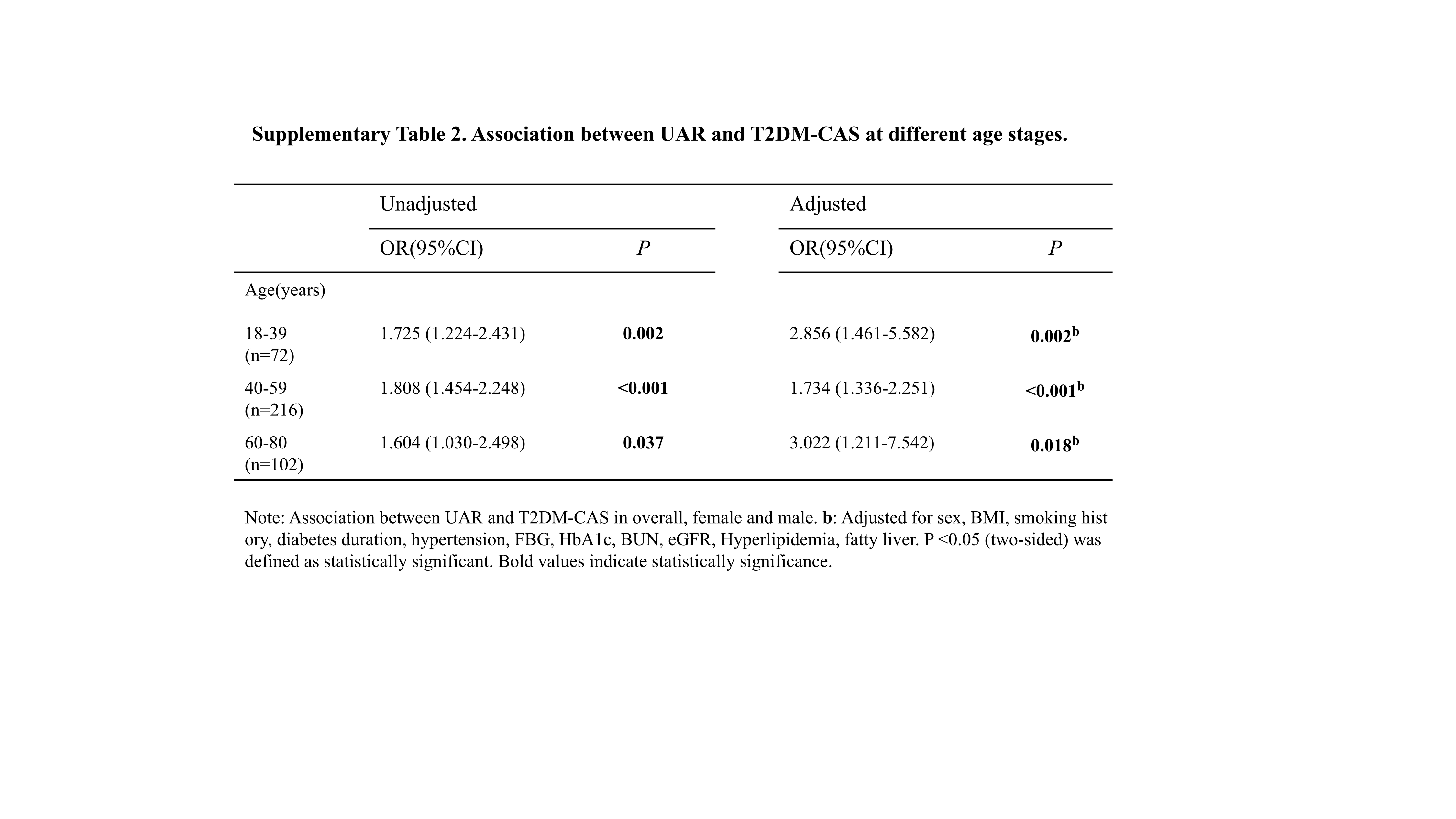

Supplement: S2 Fig — (TIF) [file pone.0320738.s003.tif]
