# Supplementary material for: Predictive value of uric acid to albumin ratio for carotid atherosclerosis in type 2 diabetes mellitus: A retrospective study
Source: PLoS One. 2025 Mar 28;20(3):e0320738. doi: 10.1371/journal.pone.0320738 (PMC11952251; doi:10.1371/journal.pone.0320738)
Supplement: Attachment — Submitted filename: Responses letter.pdf [file pone.0320738.s006.pdf]

16-February-2025

Journal: PLOS ONE

Article type: Research Article

Manuscript Number: PONE-D-24-50053

Manuscript title: Predictive Value of Uric Acid to Albumin Ratio for Carotid Atherosclerosis in Type 2 Diabetes Mellitus: A Retrospective Study

Authors: Yao Yin, Liyin Zhang, Jiaoyue Zhang and Si Jin

Dear Editor and Reviewers,

I hope this message finds you well. On behalf of my co-authors, I would like to express our sincere gratitude for the valuable feedback you and the reviewers provided on our manuscript titled "Predictive Value of Uric Acid to Albumin Ratio for Carotid Atherosclerosis in Type 2 Diabetes Mellitus: A Retrospective Study". We deeply appreciate the time and effort that you and the reviewers have dedicated to reviewing our work.

We have carefully considered all the comments and suggestions provided by you and the reviewers. Below, we outline our responses to each point and describe the corresponding changes made to the manuscript. To facilitate clarity, we have highlighted our responses in **blue** while keeping your comments in **black** in the response letter.

Thank you again for your constructive comments on our manuscript. We have carefully addressed all the points raised and ensured that the revised manuscript complies with your journal's requirements. Our detailed responses are as follows:

Academic Editor Comments and author responses:

1. comment: Please ensure that your manuscript meets PLOS ONE's style requirements, including those for file naming. The PLOS ONE style templates can be found at https://journals.plos.org/plosone/s/file?id=wjVg/PLOSOne_formatting_sample_main_body.pdf

and

https://journals.plos.org/plosone/s/file?id=ba62/PLOSOne_formatting_sample_title_authors_affiliations.pdf

1. Reply: Thank you for your reminder. I have carefully reviewed PLOS ONE's style

requirements and ensured that my manuscript is formatted according to the guidelines provided.

2. Comment: Thank you for stating the following financial disclosure:
"This work was supported by the National Natural Science Foundation of China (Grant No. 82070862, No.82370840) and the National Key R&D Program of China (Grant No. 2020YFC2008900). These funds were obtained by Professor Jin."
Please state what role the funders took in the study. If the funders had no role, please state: "The funders had no role in study design, data collection and analysis, decision to publish, or preparation of the manuscript."
If this statement is not correct you must amend it as needed.
Please include this amended Role of Funder statement in your cover letter; we will change the online submission form on your behalf.
2. Reply: Thank you for your feedback regarding the financial disclosure. We recognize the importance of clarifying the role of the funders in our study. I have added the role of the funder, Professor Jin, in the Funding Statement section of the manuscript and have also refined the CRediT authorship contribution statement. As requested, we will include this amended Role of Funder statement in our cover letter. Thank you once again for your guidance.

3. Comment: In the online submission form, you indicated that [The datasets used and analyzed during the current study are available from the corresponding author upon reasonable request].
All PLOS journals now require all data underlying the findings described in their manuscript to be freely available to other researchers, either 1. In a public repository, 2. Within the manuscript itself, or 3. Uploaded as supplementary information.
This policy applies to all data except where public deposition would breach compliance with the protocol approved by your research ethics board. If your data cannot be made publicly available for ethical or legal reasons (e.g., public availability would compromise patient privacy), please explain your reasons on resubmission and your exemption request will be escalated for approval.
3. Reply: Thank you for your guidance regarding data availability. I will upload all data underlying the findings described in the manuscript as supplementary information (S1

Dataset). This will ensure that the data is freely accessible to other researchers, in compliance with PLOS journals' policies. Thank you again for your assistance.

4. Comment: Your ethics statement should only appear in the Methods section of your manuscript. If your ethics statement is written in any section besides the Methods, please move it to the Methods section and delete it from any other section. Please ensure that your ethics statement is included in your manuscript, as the ethics statement entered into the online submission form will not be published alongside your manuscript.

4. Reply: Thank you for your valuable feedback on the ethics statement placement. We have revised the manuscript to include the ethics statement only in the Methods section and removed it from all other sections to comply with your guidelines.

5. Comment: Please review your reference list to ensure that it is complete and correct. If you have cited papers that have been retracted, please include the rationale for doing so in the manuscript text, or remove these references and replace them with relevant current references. Any changes to the reference list should be mentioned in the rebuttal letter that accompanies your revised manuscript. If you need to cite a retracted article, indicate the article's retracted status in the References list and also include a citation and full reference for the retraction notice.

5. Reply: Thank you very much for your valuable feedback regarding our reference list. We have thoroughly reviewed the references to ensure that they are complete and accurate. We do not cite any retracted papers, and all references have been updated using the PLOS ONE style in EndNote. An example of the references is provided below:

1. Zimmet P, Alberti KG, Shaw J. Global and societal implications of the diabetes epidemic. Nature. 2001;414(6865):782-7. Epub 2001/12/14. doi: 10.1038/414782a. PubMed PMID: 11742409.
2. The Lancet Diabetes E. Diabetes-related complications: a toll too high. The lancet Diabetes & endocrinology. 2024;12(9):601. Epub 2024/08/12. doi: 10.1016/s2213-8587(24)00246-8. PubMed PMID: 39128470.

Responses to reviews

Reviewer # 1:

I have reviewed the manuscript entitled 'Predictive Value of Uric Acid to Albumin Ratio for Carotid Atherosclerosis in Type 2 Diabetes Mellitus: A retrospective study'.

The manuscript sounds scientific and is well-presented. However several issues should be evaluated in order to provide more precise deductions.

The title was written twice please check.

In the introduction section the role of uric acid/ albumin should be mentioned with recent investigations citing 'The Association of Serum Uric Acid/Albumin Ratio with No-Reflow in Patients with ST Elevation Myocardial Infarction', 'Predictive value of uric acid/albumin ratio for the prediction of new-onset atrial fibrillation in patients with ST-Elevation myocardial infarction' and 'Association of Uric Acid Albumin Ratio with Recurrence of Atrial Fibrillation after Cryoballoon Catheter Ablation'. These articles will emphasize the concomitancy of inflammation and uric acid/ albumin.

Please explain the inclusion and exclusion criteria more in detail.

Please emphasize the hypothesis of the study.

The role of albumin should also be mentioned since it has several predictive effects in addition to other parameters such as in Naples score, PNI and others. Please mention the importance of albumin citing 'Overlap Between Nutritional Indices in Patients with Acute Coronary Syndrome: A Focus on Albumin' and 'Evaluation of Naples Score for Long-Term Mortality in Patients With ST-Segment Elevation Myocardial Infarction Undergoing Primary Percutaneous Coronary Intervention'.

1. Comment: The manuscript sounds scientific and is well-presented.

1. Reply: Thank you very much indeed for your comments. We are heartened by your positive remarks on our manuscript, which have reinforced our belief in the value of our research.

2. Comment: The title was written twice please check.

2. Reply: Thank you for bringing the issue of the repeated title to our attention. I sincerely apologize for my carelessness. We have corrected this and ensured that the title appears only once in the manuscript. We appreciate your careful review.

3. Comment: In the introduction section the role of uric acid/ albumin should be mentioned with recent investigations citing 'The Association of Serum Uric Acid/Albumin Ratio with No-Reflow in Patients with ST Elevation Myocardial Infarction', 'Predictive value of uric acid/albumin ratio for the prediction of new-onset atrial fibrillation in patients with ST-Elevation myocardial infarction' and 'Association of Uric Acid Albumin Ratio with Recurrence of Atrial Fibrillation after Cryoballoon Catheter Ablation'. These articles will emphasize the concomitancy of inflammation and uric acid/ albumin.

3. Reply: Thank you very much indeed for your comments. We appreciate your suggestion regarding the role of uric acid/albumin in the introduction. In response, we have revised the introduction to include a discussion of recent studies you recommended in lines 91-96, highlighting the importance of the uric acid/albumin ratio and emphasizing its relationship with inflammation.

as follows:

nutrition, inflammation, and metabolic syndrome. Multiple studies have highlighted the close association between inflammation and the UAR. Research indicates that UAR is an independent risk factor for new-onset atrial fibrillation (AF) in patients with STEMI[15] and is also independently associated with the recurrence of AF after catheter ablation[16]. In STEMI patients undergoing percutaneous coronary intervention (PCI), UAR is independently related to no-reflow phenomena[17].

the references cited are as follows:

15. Selçuk M, Çınar T, Şaylık F, Akbulut T, Asal S, Çiçek V, et al. Predictive value of uric acid/albumin ratio for the prediction of new-onset atrial fibrillation in patients with ST-Elevation myocardial infarction. Revista de investigacion clinica; organo del Hospital de Enfermedades de la Nutricion. 2022;74(3):156-64. Epub 2022/07/08. doi: 10.24875/ric.22000072. PubMed PMID: 35797660.

16. Karataş MB, Durmuş G, Zengin A, Gökalp M, Hayıroğlu M, Çınar T, et al. Association of Uric Acid Albumin Ratio with Recurrence of Atrial Fibrillation after Cryoballoon Catheter Ablation. Medicina (Kaunas, Lithuania). 2022;58(12). Epub 2022/12/24. doi: 10.3390/medicina58121872. PubMed PMID: 36557074; PubMed Central PMCID: PMCPMC9784806.

17. Çınar T, Şaylık F, Hayıroğlu M, Asal S, Selçuk M, Çiçek V, et al. The Association of Serum Uric Acid/Albumin Ratio with No-Reflow in Patients with ST Elevation Myocardial Infarction. Angiology. 2023;74(4):381-6. Epub 2022/06/22. doi: 10.1177/00033197221110700. PubMed PMID: 35726733.

4. Comment: Please explain the inclusion and exclusion criteria more in detail.

4. Reply: Thank you very much for your comments. We appreciate your request for a more detailed explanation of the inclusion and exclusion criteria. In response, we have expanded the Study Population section of the manuscript to provide greater clarity on these criteria. Specifically, we have elaborated on the inclusion and exclusion criteria in lines 116-117 and 128-134.

as follows:

The inclusion criteria were patients aged 18-79 years with a confirmed diagnosis of T2DM. Our exclusion criteria were as follows: (1) Undefined or suspected non-type 2

10^9 cells/L) or leukopenia (< 4 × 10^9 cells/L); (11) Incomplete clinical data. By excluding patients with non-type 2 diabetes, acute complications, and other severe comorbidities, we ensured the homogeneity of the study population. Additionally, the exclusion of patients with tumors, pancreatic diseases, and autoimmune disorders minimized the potential confounding effects of these conditions on metabolic parameters. These exclusion criteria were designed to enhance the accuracy of the analysis and the reliability of the study conclusions.

5. Comment: Please emphasize the hypothesis of the study.

5. Reply: Thank you for your valuable suggestion to highlight the study's hypothesis. In response, we have revised the manuscript to present a clearer and more prominent statement of our hypothesis. This has been incorporated into the introduction section to ensure it stands out and effectively conveys the key objectives of our research. You can find the revised text in lines 103-106 of the introduction section.

as follows:

T2DM-CAS. Therefore, we hypothesize that UAR may serve as a predictive biomarker for T2DM-CAS. This study aims to explore the potential of UAR as a novel biomarker for the early screening and intervention of T2DM-CAS, which could contribute to reducing the risk of further vascular complications.

6. Comment: The role of albumin should also be mentioned since it has several predictive effects in addition to other parameters such as in Naples score, PNI and others. Please mention the importance of albumin citing 'Overlap Between Nutritional Indices in Patients with Acute Coronary Syndrome: A Focus on Albumin' and 'Evaluation of Naples Score for Long-Term Mortality in Patients With ST-Segment Elevation Myocardial Infarction Undergoing Primary Percutaneous Coronary Intervention'.

6. Reply: Thank you very much indeed for your comments. Thank you for your insightful comment regarding the role of albumin. We recognize its significant predictive effects in various clinical parameters, including the Naples score and the Prognostic Nutritional Index (PNI). In response, we have revised the manuscript to include a discussion on the importance of albumin in lines 83-89 of the introduction.

as follows:

progression of atherosclerosis. ALB also plays a role in regulating endothelial function and vascular tone. A decrease in its levels can impair the bioavailability of nitric oxide, leading to vasoconstriction, increased vascular resistance, and impaired coronary perfusion, thereby making patients more susceptible to ischemic events[13]. Additionally, albumin-based indicators, such as the Naples Prognostic Score (NPS) and the Prognostic Nutritional Index (PNI), have demonstrated significant predictive value[14].↵

the references cited are as follows:

13. Hayıroğlu M, Altay S. Overlap Between Nutritional Indices in Patients with Acute Coronary Syndrome: A Focus on Albumin. Balkan medical journal. 2024;41(5):324-5. Epub 2024/08/01. doi: 10.4274/balkanmedj.galenos.2024.190724. PubMed PMID: 39086257; PubMed Central PMCID: PMCPMC11588916.↵

14. Şaylık F, Çınar T, Selçuk M, Akbulut T, Hayıroğlu M, Tanboğa İ H. Evaluation of Naples Score for Long-Term Mortality in Patients With ST-Segment Elevation Myocardial Infarction Undergoing Primary Percutaneous Coronary Intervention. Angiology. 2024;75(8):725-33. Epub 2023/04/15. doi: 10.1177/00033197231170982. PubMed PMID: 37058422.↵

Reviewer # 2:

1. Comment: The authors should consistently use either sex or Gender in every places.

1. Reply: Thank you for your valuable feedback regarding the use of terminology. We apologize for this oversight and recognize the importance of maintaining consistency in the manuscript. In response, we have reviewed the text and replaced all instances of "sex" with "gender" to ensure consistency throughout the manuscript. Thank you again for your constructive reminder.

2. Comment: In the table, "n" should be used instead of "N". “N” denotes population.

2. Reply: Thank you for your comment regarding the notation in the table. We appreciate your attention to detail. In response, we have revised the table to use "n" instead of "N," as "N" typically denotes the population. This change enhances the accuracy of our data presentation.

3. Comment: Kindly provide a more detailed description of Figures 1, 2 and 3.

3. Reply: Thank you for your constructive feedback regarding the figures. We appreciate your suggestion for more detailed descriptions. In response, we have enhanced the descriptions of Figures 1, 2, and 3 in the manuscript to provide clearer insights into the data presented.

the details are as follows:

Fig 1. Distribution of UAR between the WCAS and CAS group.

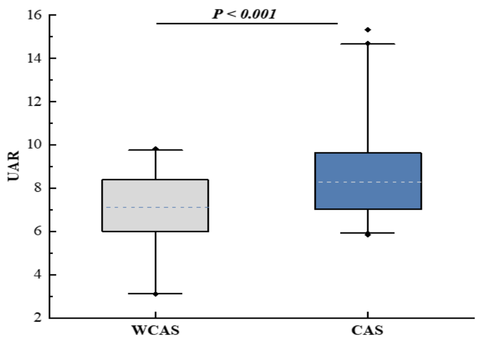

The box plot in Figure 1 shows that UAR in the CAS group was significantly higher than that in the WCAS group ($P < 0.001$). On the box plots, central lines represent the median, the length of the box represents the interquartile range and the lines extend to minimum and maximum values. $P < 0.05$ (two-sided) was defined as statistically significant.

Fig 2. Distribution of UAR Across Different Groups.

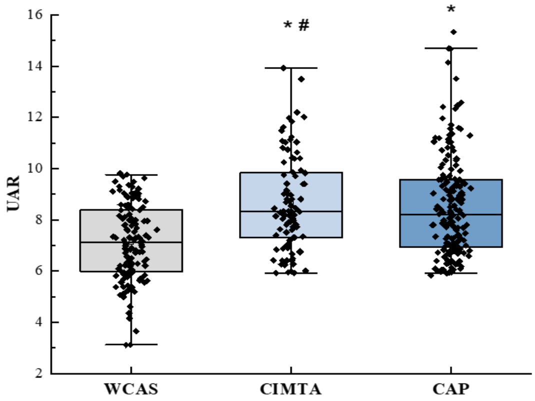

The box plot in the figure 2 displays the median as the central line, the interquartile range as the length of the box, and the lines extending to the minimum and maximum values. * P < 0.05 versus WCAS; # P > 0.05 versus CAP. $P < 0.05$ (two-sided) was defined as statistically significant.

Fig 3. Distribution of UAR between the SC and MC group.

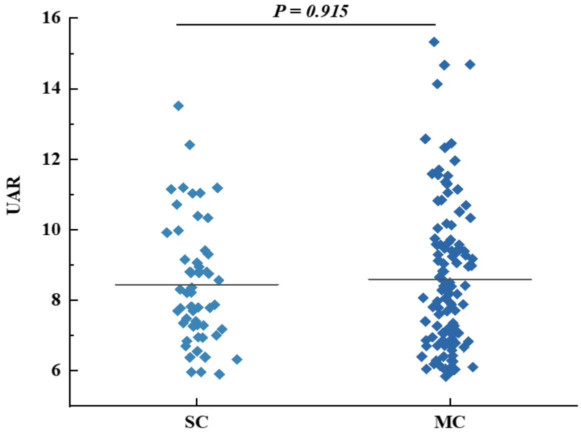

The differences in UAR between the SC and MC groups. In the scatter plot, the data were presented as median (min -max). $P < 0.05$ (two-sided) was defined as statistically significant.

4. Comment: The authors have not addressed the topic of women patients, and they have not even mentioned it in the limitations section.

4. Reply: Thank you for your insightful comment regarding the inclusion of female patients in our study. We acknowledge the importance of considering gender differences

in clinical research. We have added the results of the gender-stratified analysis and discussed them in lines 420-426 of the discussion section in the Revised Manuscript with Track Changes (in lines 417-423 of the final Manuscript). And indeed, after adjusting for confounding factors, it was found that female patients with high UAR levels have a higher risk of disease. Thank you once again for your profound insights. Your input has helped us enhance the comprehensiveness of our manuscript.

as follows:

~~significant positive correlation between UAR and BMI.~~ We also observed a significant positive correlation between UAR and both gender and BMI. Through gender-stratified analysis (see S1 Fig), the results indicated that UAR was significantly associated with outcomes in both male and female patients. However, after adjusting for potential confounders, the risk increase was more pronounced in females. This finding highlights UAR as an important risk factor and suggests a significant gender-based difference in its impact. Obesity is a metabolic disease that induces insulin resistance by altering the

S1 Fig is as follows:

Supplementary Table 1. Association between UAR and T2DM-CAS in overall, female and male.

	Unadjusted		Adjusted	
	OR(95%CI)	*P*	OR(95%CI)	*P*
Total UAR categories (n=390)	1.691 (1.448-1.975)	**<0.001**	1.895 (1.499-2.307)	**<0.001[a]**
Female UAR categories (n=113)	1.709 (1.287-2.270)	**<0.001**	2.562 (1.439-4.561)	**0.001[a]**
Male UAR categories (n=277)	1.675 (1.389-2.019)	**<0.001**	1.898 (1.463-2.462)	**<0.001[a]**

Note: Association between UAR and T2DM-CAS in overall, female and male. **a**: Adjusted for age, BMI, diabetes duration, hypertension, FBG, HbA1c, TG, TC, HDL-C, LDL-C, BUN, Cr, eGFR, Hyperlipidemia, fatty liver. P <0.05 (two-sided) was defined as statistically significant. Bold values indicate statistically significance.

Reviewer # 3:

After reviewing the manuscript, I would like to highlight several points as drawbacks
*The study's sample size may limit the generalizability of its findings, as it is confined to a particular demographic.
*The retrospective nature of the study introduces potential biases and limits the capacity to infer causality.
As recommendations, details on how carotid IMT and plaques were measured could be clarified to ensure reproducibility. Additionally, addressing potential confounding variables in analyses would strengthen the conclusions drawn.
However, The study investigates the uric acid to albumin ratio (UAR) as a predictive biomarker for carotid atherosclerosis specifically in patients with type 2 diabetes mellitus (T2DM). This offers new insights, particularly since UAR combines elements of both nutritional status and inflammatory response.

1. Comment: Details on how carotid IMT and plaques were measured could be clarified to ensure reproducibility.
1. Reply: Thank you for your valuable comments regarding the measurement methods for carotid intima-media thickness (IMT) and plaques. We agree that a clear description of these methods is essential for ensuring reproducibility. In response, we have provided more detailed descriptions of the ultrasound probe frequency, patient positioning, and specific examination sites, which have been added to lines 155-159 and 162-166 of the Revised Manuscript with Track Changes (155-159 and 160-164 of the final Manuscript). We appreciate your feedback once again.
as follows:

diagnostic instrument by trained and certified ultrasound specialists. Imaging was performed using the same color Doppler device equipped with a 7.0–10 MHz transducer. All patients were placed in a supine position, with their heads slightly turned toward the opposite side of the examination and tilted backward to fully expose the carotid arteries.↵

~~carotid artery.~~ Bilateral assessments of the common carotid artery (CCA) were performed both transversely and longitudinally, including the proximal CCA, distal CCA, bulb, internal carotid artery (ICA), and external carotid artery (ECA). Scanning was conducted in various directions to evaluate atherosclerotic plaques and stenosis, as well as to measure intima-media thickness (IMT). The carotid intima-media thickness

2. Comment: Addressing potential confounding variables in analyses would strengthen the conclusions drawn.

2. Reply: Thank you for your insightful comment regarding the consideration of potential confounding variables in our analyses. We recognize that addressing these variables is crucial for strengthening the validity of our conclusions. In response, we conducted stratified analyses based on gender and age, and we have discussed these findings in lines 420-426 and 440-445 of the Discussion section in the Revised Manuscript with Track Changes (in lines 417-423 and 437-442 of the final Manuscript). In addition, we have further explored this topic in the limitations section, incorporating your valuable comments.

the discussion on gender stratified analysis is as follows:

~~significant positive correlation between UAR and BMI.~~ We also observed a significant positive correlation between UAR and both gender and BMI. Through gender-stratified analysis (see S1 Fig), the results indicated that UAR was significantly associated with outcomes in both male and female patients. However, after adjusting for potential confounders, the risk increase was more pronounced in females. This finding highlights UAR as an important risk factor and suggests a significant gender-based difference in its impact. Obesity is a metabolic disease that induces insulin resistance by altering the

the discussion on age stratified analysis is as follows:

studies[50]. After performing a stratified analysis of age in this study (see S2 Fig), it was found that the association between UAR and T2DM-CAS was significant across different age groups. Among all age groups, the 60-80 age group showed a significant increase in odds ratio after controlling for potential confounding factors. This suggests that special attention should be given to treatment strategies for the elderly population in clinical applications. ROC curve analysis demonstrated that UAR effectively predicts

The additional limitations are as follows:

This study also has several limitations. First, it is a retrospective cross-sectional study conducted at a single center, which prevents us from establishing a causal relationship between UAR and T2DM-CAS. Secondly, our sample size is relatively small, and we only investigated this relationship in T2DM subjects, which may lead to less precise estimates and limit the generalizability of our findings. Therefore, the results need to be validated in other types of diabetes and in healthy subjects. Additionally, in this study, we could not completely rule out other potential confounding factors. For example, we did not conduct stratified analyses based on the use of hypoglycemic agents, education level, social status, and economic level between the two groups of patients, nor did we incorporate psychological factors into the analysis. Third, the hematological parameters

S1 Fig. and S2 Fig are as follows:

Supplementary Table 1. Association between UAR and T2DM-CAS in overall, female and male.

	Unadjusted		Adjusted	
	OR(95%CI)	*P*	OR(95%CI)	*P*
Total UAR categories (n=390)	1.691 (1.448-1.975)	**<0.001**	1.895 (1.499-2.307)	**<0.001[a]**
Female UAR categories (n=113)	1.709 (1.287-2.270)	**<0.001**	2.562 (1.439-4.561)	**0.001[a]**
Male UAR categories (n=277)	1.675 (1.389-2.019)	**<0.001**	1.898 (1.463-2.462)	**<0.001[a]**

Note: Association between UAR and T2DM-CAS in overall, female and male. **a**: Adjusted for age, BMI, diabetes duration, hypertension, FBG, HbA1c, TG, TC, HDL-C, LDL-C, BUN, Cr, eGFR, Hyperlipidemia, fatty liver. P <0.05 (two-sided) was defined as statistically significant. Bold values indicate statistically significance.

Supplementary Table 2. Association between UAR and T2DM-CAS at different age stages.

	Unadjusted		Adjusted	
	OR(95%CI)	*P*	OR(95%CI)	*P*
Age(years)				
18-39 (n=72)	1.725 (1.224-2.431)	**0.002**	2.856 (1.461-5.582)	**0.002[b]**
40-59 (n=216)	1.808 (1.454-2.248)	**<0.001**	1.734 (1.336-2.251)	**<0.001[b]**
60-80 (n=102)	1.604 (1.030-2.498)	**0.037**	3.022 (1.211-7.542)	**0.018[b]**

Note: Association between UAR and T2DM-CAS in overall, female and male. **b**: Adjusted for sex, BMI, smoking history, diabetes duration, hypertension, FBG, HbA1c, BUN, eGFR, Hyperlipidemia, fatty liver. P <0.05 (two-sided) was defined as statistically significant. Bold values indicate statistically significance.

3. Comment: However, the study investigates the uric acid to albumin ratio (UAR) as a predictive biomarker for carotid atherosclerosis specifically in patients with type 2 diabetes mellitus (T2DM). This offers new insights, particularly since UAR combines elements of both nutritional status and inflammatory response.

3. Reply: Thank you for your thoughtful comments. We greatly appreciate your recognition of our study on the uric acid to albumin ratio (UAR) as a predictive biomarker for carotid atherosclerosis (CAS) in type 2 diabetes mellitus (T2DM). Your insights regarding the interplay of nutritional status and inflammatory response are invaluable to us. Thank you for helping me refine my manuscript to a higher standard.

At last, I sincerely thank all of you once again for your constructive feedback, which undoubtedly helps enhance the quality and clarity of our manuscript.

Best wishes.

Sincerely,

Corresponding author Si Jin
