# Supplementary material for: Predictive value of uric acid to albumin ratio for carotid atherosclerosis in type 2 diabetes mellitus: A retrospective study
Source: PLoS One. 2025 Mar 28;20(3):e0320738. doi: 10.1371/journal.pone.0320738 (PMC11952251; doi:10.1371/journal.pone.0320738)
Supplement: Attachment — Submitted filename: Review comment PLOS.docx [file pone.0320738.s004.docx]

1. The authors should consistently use either sex or Gender in every places.

2. In the table, "n" should be used instead of "N". “N” denotes population.

3. Kindly provide a more detailed description of Figures 1, 2 and 3.

4. The authors have not addressed the topic of women patients, and they have not even mentioned it in the limitations section.
